# Supplementary material for: Coxiella burnetii manipulates the lysosomal protease cathepsin B to facilitate intracellular success
Source: Nat Commun. 2025 Apr 24;16:3844. doi: 10.1038/s41467-025-59283-3 (PMC12022341; doi:10.1038/s41467-025-59283-3)
Supplement: Supplementary file 1 — Supplementary Information [file 41467_2025_59283_MOESM1_ESM.pdf]

Supporting information

Coxiella burnetii **manipulates the lysosomal protease cathepsin B to facilitate intracellular success**

Lauren E. Bird[1,2], Bangyan Xu[3], Andrew D. Hobbs[2], Alexander R. Ziegler[3], Nichollas E. Scott[1], Patrice Newton[1,2], David R. Thomas[2], Laura E. Edgington-Mitchell[3,#,*], Hayley J. Newton[1,2,#,*]

[1] Department of Microbiology and Immunology at the Peter Doherty Institute for Infection and Immunity, The University of Melbourne, Melbourne, VIC 3000, Australia

[2] Infection Program, Monash Biomedicine Discovery Institute, Department of Microbiology, Monash University, VIC 3800, Australia

[3] Department of Biochemistry and Pharmacology at the Bio21 Molecular Science and Biotechnology Institute, The University of Melbourne, VIC 3000, Australia

[#] These authors jointly supervised this work

*** Corresponding authors:**
Laura E. Edgington-Mitchell
Email: laura.edgingtonmitchell@unimelb.edu.au
Hayley J. Newton
Email: hayley.newton@monash.edu

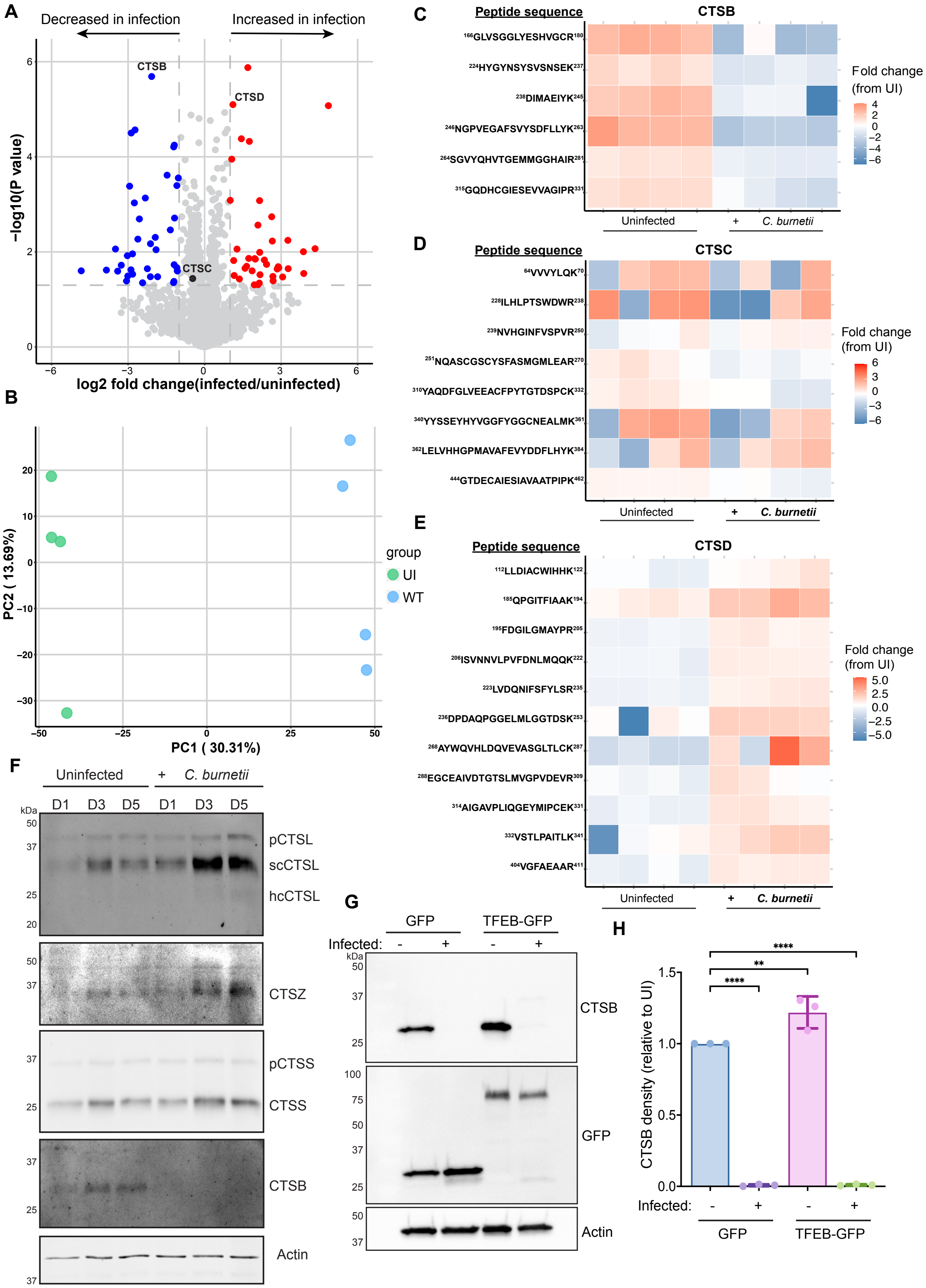
A
Decreased in infection
Increased in infection
CTSB
CTSD
CTSC
−log10(P value)
log2 fold change(infected/uninfected)
B
PC2 (13.69%)
PC1 (30.31%)
group
UI
WT
C
Peptide sequence
CTSB
[166]GLVSGGLYESHVGCR[180]
[224]HYGYNSYSVSNSEK[237]
[238]DIMAEIYK[245]
[246]NGPVEGAFSVYSDFLLYK[263]
[264]SGVYQHVTGEMMGGHAIR[281]
[315]GQDHCGIESEVVAGIPR[331]
Fold change
(from UI)
Uninfected
+ C. burnetii
D
Peptide sequence
CTSC
[64]VVVYLQK[70]
[228]ILHLPTSWDWR[238]
[239]NVHGINFVSPVR[250]
[251]NQASCGSCYSFASMGMLEAR[270]
[310]YAQDFGLVEEACFPYTGTDSPCK[332]
[340]YYSSEYHYVGGFYGGCNEALMK[361]
[362]LELVHHGPMAVAFEVYDDFLHYK[384]
[444]GTDECAIESIAVAATPIPK[462]
Fold change
(from UI)
Uninfected
+ C. burnetii
E
Peptide sequence
CTSD
[112]LLDIACWIHHK[122]
[185]QPGITFIAAK[194]
[195]FDGILGMAYPR[205]
[206]ISVNNVLPVFDNLMQQK[222]
[223]LVDQNIFSFYLSR[235]
[236]DPDAQPGGELMLGGTDSK[253]
[268]AYWQVHLDQVEVASGLTLCK[287]
[288]EGCEAIVDTGTSLMVGPVDEVR[309]
[314]AIGAVPLIQGEYMIPCEK[331]
[332]VSTLPAITLK[341]
[404]VGFAEAAR[411]
Fold change
(from UI)
Uninfected
+ C. burnetii
F
Uninfected
+ C. burnetii
D1 D3 D5 D1 D3 D5
kDa
pCTSL
scCTSL
hcCTSL
CTSZ
pCTSS
CTSS
CTSB
Actin
G
GFP
TFEB-GFP
Infected: - + - +
kDa
CTSB
GFP
Actin
H
CTSB density (relative to UI)
Infected: - + - +
GFP
TFEB-GFP
****
**
****

Supplementary figure 1. Mass spectrometry reveals that cathepsin B is lost during *C. burnetii* infection of THP-1 cells. (A) Scatter plot depicting changes to THP-1 host proteome following infection with *C. burnetii* (WT) for 3 days. UI n=4, WT n=4. Horizontal axis represents fold change (infected/uninfected), while vertical axis shows statistical significance as determined by student's t-test. Proteins with -log*(p)* > 1.3 and fold change > 1/-1 are coloured. Proteins of interest to this study (CTSB, CTSC, CTSD) are labelled. Horizontal dashed line denotes significance cut off ($p < 0.05$), vertical dashed line represents fold change > 1. **(B)** Principal component analysis (PCA) plot of proteomics data in (A) was performed to visualise clustering of experimental groups. Uninfected (UI) data are shown in green, *C. burnetii*-infected (WT) data are shown in blue (n=4/group). **(C-E)** Heatmaps of Z-scored max label-free quantification (LFQ) intensity values for peptides identified in CTSB (C), CTSC (D), and CTSD (E). Each column represents a biological replicate (n=4/group). Plots were generated in *R* (v4.1.2) using ggplot2. **(F)** Immunoblot on THP-1 lysates following infection with *C. burnetii* (MOI 25). pCTSL = pro-cathepsin L, scCTSL = single chain cathepsin L, hcCTSL = heavy chain cathepsin L, CTSZ = cathepsin Z, pCTSS = pro-cathepsin S, CTSB = cathepsin B **(G)** Immunoblot on THP-1 lysates from cells stably expressing GFP alone or TFEB-GFP. Cells were infected with *C. burnetii* for 72h (MOI 25) before immunoblotting. Blot is representative of 3 independent experiments, quantified in **(H).** Statistical significance was determined using a one-way ANOVA with Dunnett's post hoc test. **** = $p < 0.0001$, ** = $p < 0.01$. Error bars represent standard deviation. Mass spectrometry source data are available via ProteomeXchange with identifier PXD052888.

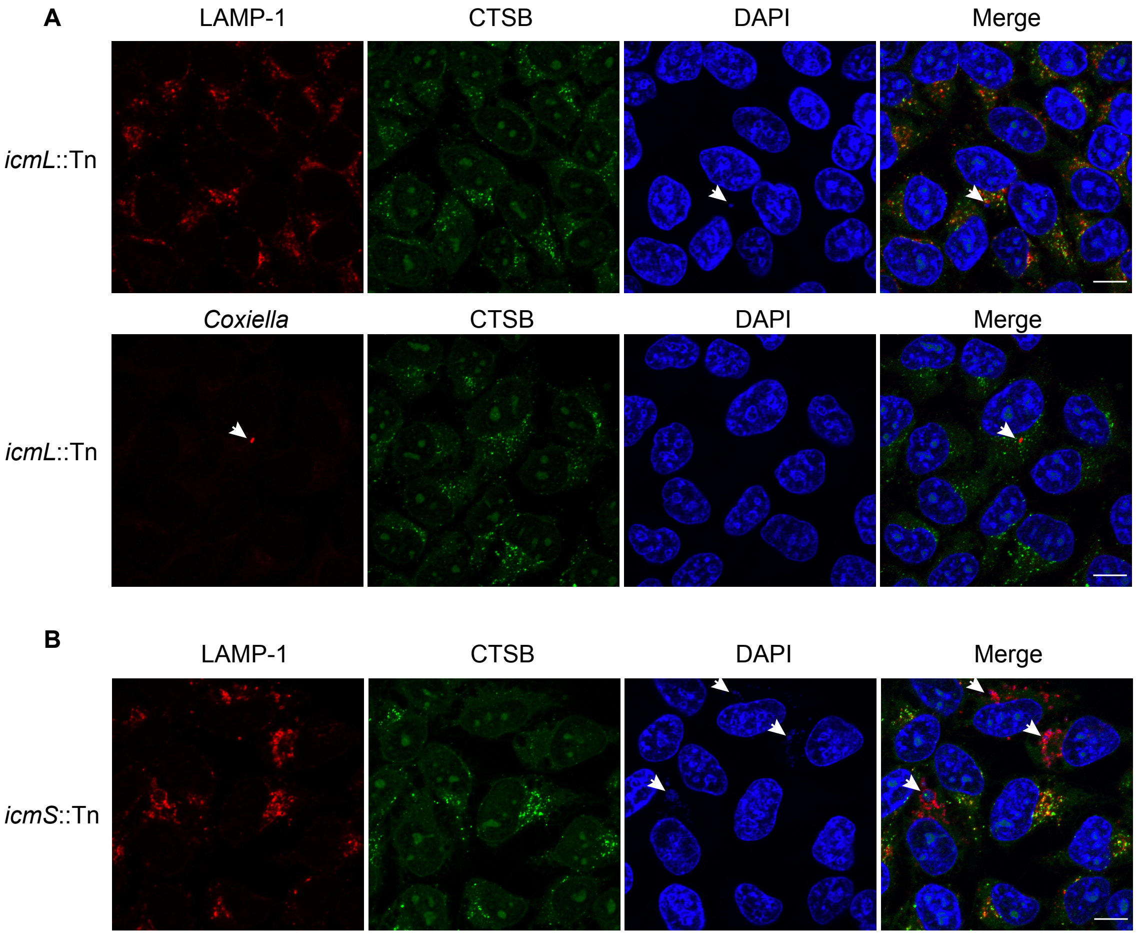

Supplementary figure 2. Microscopic analysis of cells infected with *C. burnetii* Dot/Icm mutants. (A) HeLa cells were infected with *C. burnetii icmL*::Tn at a MOI of 10 for 72 h before being fixed and stained with antibodies to cathepsin B (CTSB, green), LAMP-1 (red, top panel), *Coxiella* (red, bottom panel) or DAPI (blue). Bacteria are indicated with white arrowhead. Scale bar = 10 µm. **(B)** HeLa cells were infected with *C. burnetii icmS*::Tn at a MOI of 10 for 72 h before being fixed and stained with antibodies to cathepsin B (CTSB, green) and LAMP-1 (red) and DAPI (blue). Bacteria are indicated with white arrowhead. Scale bar = 10 µm.

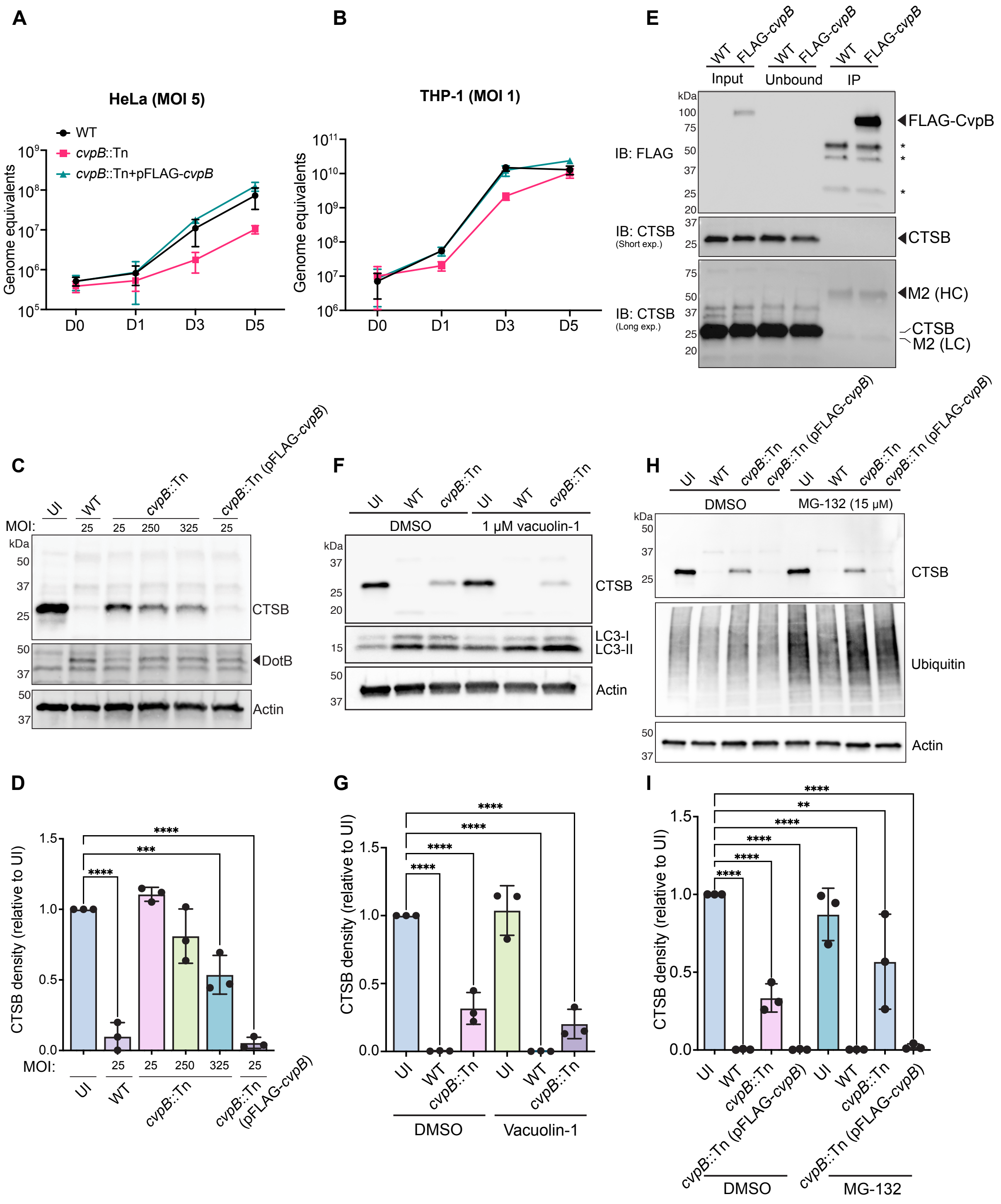
A
HeLa (MOI 5)
WT
cvpB::Tn
cvpB::Tn+pFLAG-cvpB
Genome equivalents
D0
D1
D3
D5
B
THP-1 (MOI 1)
Genome equivalents
E
WT FLAG-cvpB WT FLAG-cvpB WT FLAG-cvpB
Input Unbound IP
IB: FLAG
FLAG-CvpB
IB: CTSB (Short exp.)
CTSB
IB: CTSB (Long exp.)
M2 (HC)
CTSB
M2 (LC)
C
UI WT cvpB::Tn cvpB::Tn (pFLAG-cvpB)
MOI: 25 25 250 325 25
CTSB
DotB
Actin
F
UI WT cvpB::Tn UI WT cvpB::Tn
DMSO
1 µM vacuolin-1
CTSB
LC3-I
LC3-II
Actin
H
UI WT cvpB::Tn cvpB::Tn (pFLAG-cvpB) UI WT cvpB::Tn cvpB::Tn (pFLAG-cvpB)
DMSO
MG-132 (15 µM)
CTSB
Ubiquitin
Actin
D
CTSB density (relative to UI)
MOI:
UI WT cvpB::Tn cvpB::Tn (pFLAG-cvpB)
G
CTSB density (relative to UI)
UI WT cvpB::Tn UI WT cvpB::Tn
DMSO
Vacuolin-1
I
CTSB density (relative to UI)
UI WT cvpB::Tn cvpB::Tn (pFLAG-cvpB) UI WT cvpB::Tn cvpB::Tn (pFLAG-cvpB)
DMSO
MG-132

Supplementary figure 3. CvpB is indirectly involved in cathepsin B loss during infection. (A) HeLa cells were infected with wild-type (WT), *cvpB*::Tn or the complemented mutant (*cvpB*::Tn (pFLAG-*cvpB*) at a MOI of 5. Cells were lysed and harvested for genomic DNA extraction immediately after infection (D0) or at days 1, 3 and 5 post infection. qPCR was performed to determine genomic equivalents using primers specific to *ompA*. **(B)** As for (A) but in THP-1 cells infected at a MOI of 1. **(C, D)** THP-1 cells were left uninfected (UI) or infected with wild-type (WT), *cvpB*::Tn or *cvpB*::Tn (pFLAG-*cvpB*) at the indicated MOI. After 72 h of infection, cells were harvested for SDS-PAGE and western blotting. (D) Quantification of (C). Data reflect three independent experiments. **(E)** THP-1 cells were infected with wild-type *C. burnetii* or *cvpB*::Tn (pFLAG-*cvpB*) at a MOI of 30 for 24 h before being subject to immunoprecipitation with anti-FLAG M2 magnetic beads. M2 (HC) = heavy chain of M2 antibody; M2 (HC) = light chain of M2 antibody **(F, G)** THP-1 cells were infected with *C. burnetii* WT or *cvpB*::Tn at a MOI of 50 for 72h prior to washing and incubating in serum-free RPMI +/- 1 μM vacuolin-1. After 6h of treatment, cells were harvested for SDS-PAGE and immunoblotting to assess cathepsin B abundance. Lipidation of LC3 was used to confirm drug activity. (G) Quantification of cathepsin B density in (F). Data reflect three independent experiments. **(H, I)** THP-1 cells were infected with WT, *cvpB*::Tn or *cvpB*::Tn (pFLAG-*cvpB*) at a MOI of 25 for 72h. After this time, 15 μM MG-132 was added and left for a further 8hrs. Following this, cells were harvested for immunoblotting. Accumulation of ubiquitin was used to confirm drug activity. (I) Quantification of cathepsin B density in (H). Data reflect three independent experiments. Statistical significance between groups for all densitometry experiments was calculated using one-way ANOVA with Tukey's post hoc test. ** $p < 0021$, *** $p < 0002$, **** $p < 0001$. Error bars denote SD.

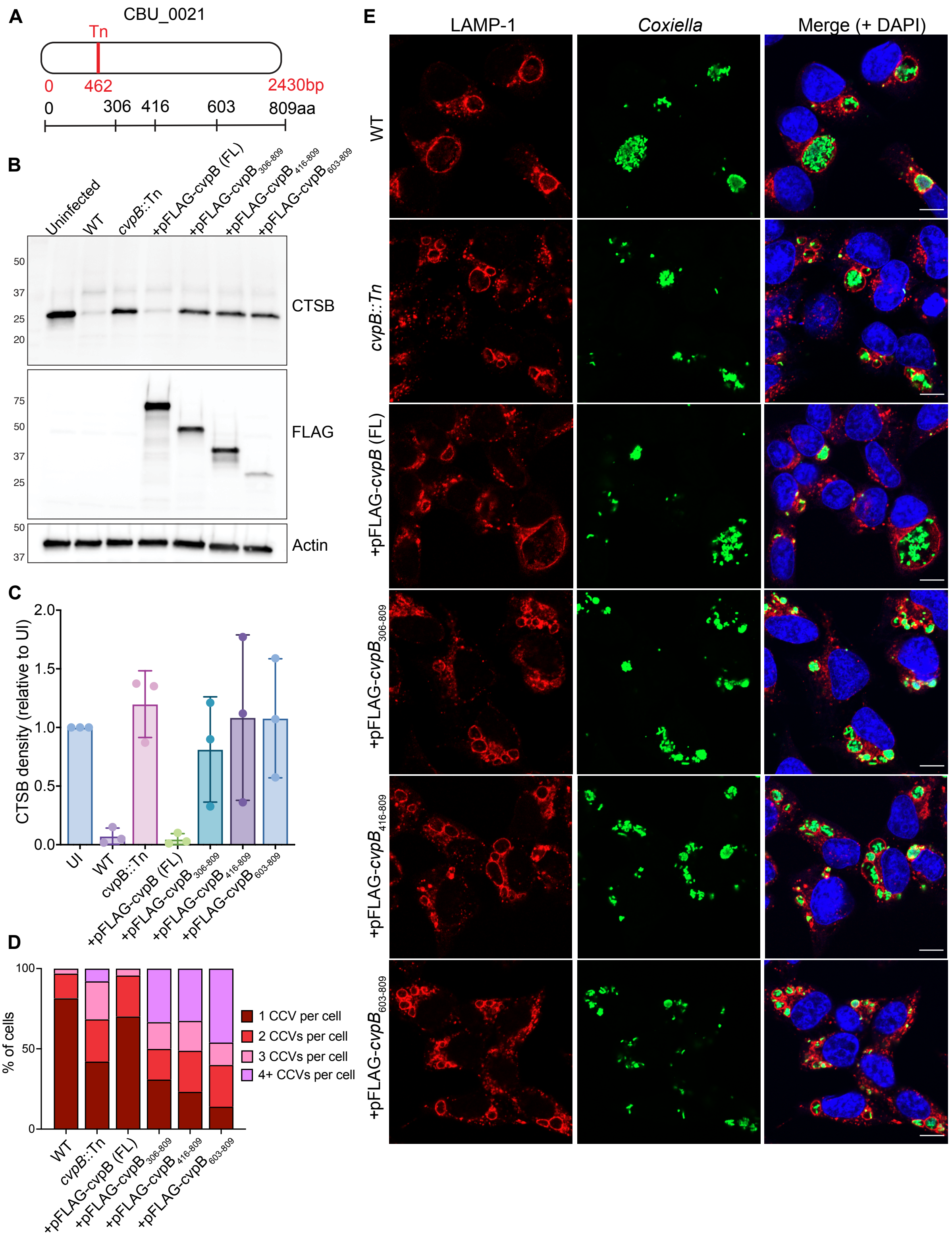
A
CBU_0021
Tn
0
462
2430bp
0
306
416
603
809aa
B
Uninfected
WT
cvpB::Tn
+pFLAG-cvpB (FL)
+pFLAG-cvpB 306-809
+pFLAG-cvpB 416-809
+pFLAG-cvpB 603-809
CTSB
FLAG
Actin
C
CTSB density (relative to UI)
UI
WT
cvpB::Tn
+pFLAG-cvpB (FL)
+pFLAG-cvpB 306-809
+pFLAG-cvpB 416-809
+pFLAG-cvpB 603-809
D
% of cells
1 CCV per cell
2 CCVs per cell
3 CCVs per cell
4+ CCVs per cell
WT
cvpB::Tn
+pFLAG-cvpB (FL)
+pFLAG-cvpB 306-809
+pFLAG-cvpB 416-809
+pFLAG-cvpB 603-809
E
LAMP-1
Coxiella
Merge (+ DAPI)
WT
cvpB::Tn
+pFLAG-cvpB (FL)
+pFLAG-cvpB 306-809
+pFLAG-cvpB 416-809
+pFLAG-cvpB 603-809

Supplementary figure 4. The N-terminus of CvpB is required for cathepsin B removal during infection through its ability to promote CCV fusion. (A) Schematic of CvpB protein. Nucleotide length and approximate position of transposon indicated in red. Black reflects amino acid length and various truncations used these experiments. **(B, C)** THP-1 cells were uninfected or infected with wild-type (WT), *cvpB*::Tn or *cvpB*::Tn complemented with various truncations of CvpB. After 72 h, cells were harvested for SDS-PAGE and immunoblotting. (C) Quantification of immunoblots in (B). Data reflect three independent experiments. **(D, E)** THP-1 cells were infected as in (B) but on glass coverslips. After 72 h of infection, cells were fixed and stained with antibodies against LAMP-1 (red) and *Coxiella* (green). DAPI (blue) was used to visualise DNA. (E) Quantification of number of CCVs per cell, presented as stacked bar chart. WT n=65, *cvpB*::Tn n=38, *cvpB*::Tn(pFLAG-*cvpB*) n=47, +pFLAG-$cvpB_{306\text{-}809}$ n=42, +pFLAG-$cvpB_{416\text{-}809}$ n=43, +pFLAG-$cvpB_{603\text{-}809}$ n=50. Scale bar = 10 µm.

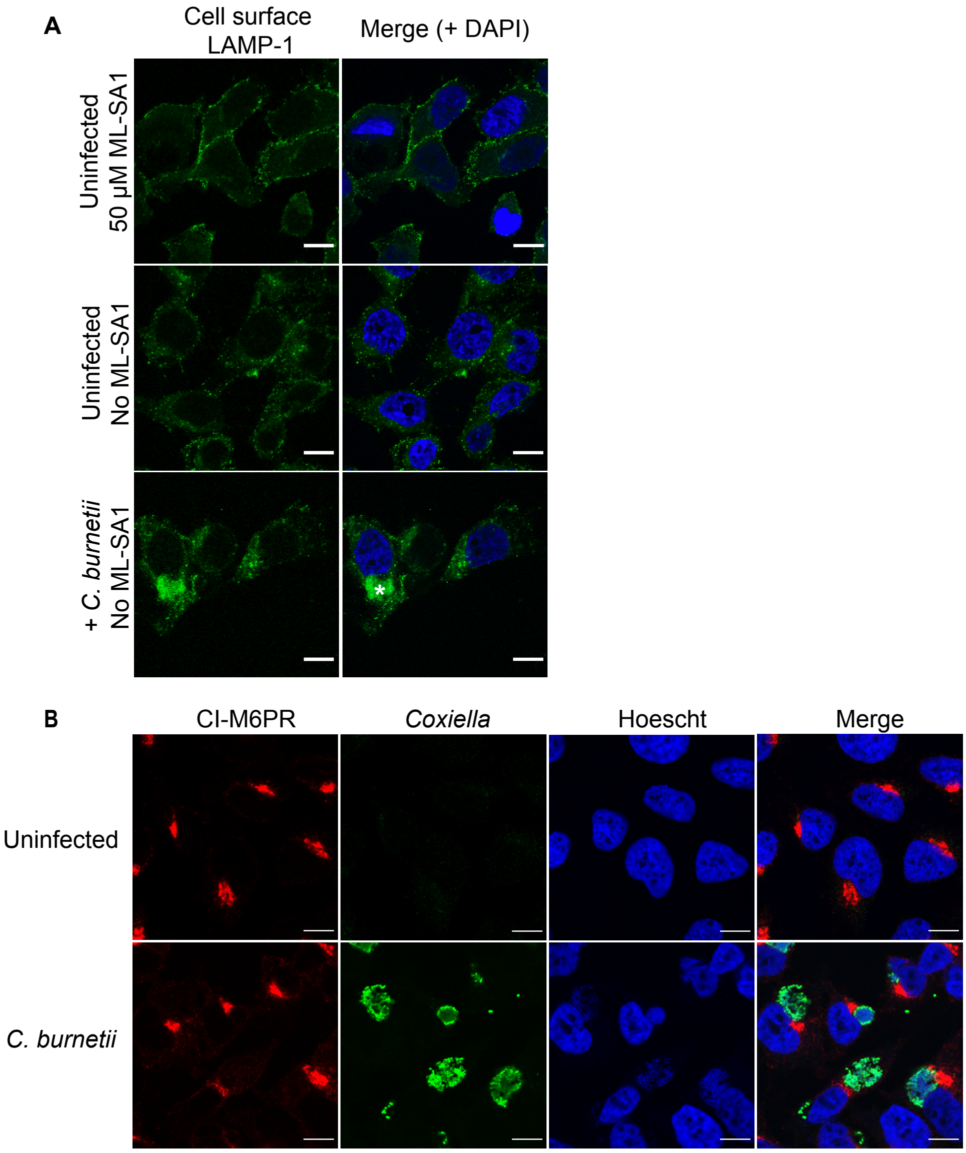

Supplementary figure 5. *C. burnetii* infection does not induce lysosomal exocytosis or defective mannose-6-phosphate trafficking. (A) HeLa cells were uninfected or infected for 3 days with *C. burnetii* (MOI 100) on glass coverslips. After this time, cells were left untreated or were treated with 50 µM ML-SA1 for 5 min to induce lysosomal exocytosis, then transferred to ice to prevent re-endocytosis of cell surface LAMP-1. Live cells were then immunostained with an antibody to the lumenal epitope of LAMP-1 before being fixed for microscopy. **(B)** HeLa cells were uninfected or infected for 3 days with WT *C. burnetii* on glass coverslips before being fixed and immunostained with antibodies to the cation-independent mannose-6-phosphate receptor (CI-M6PR) or *Coxiella*. DNA was visualised with Hoescht. Scale bar = 10 µm.

Supplementary Tables

**THP-1 lysate**						
GO cellular component:	Vacuolar lumen	Primary lysosome	Azurophil granule	Secretory granule	Cytoplasmic vesicle	Vesicle lumen
Enrichment ratio	14.095	12.731	12.731	9.25	9.1641	9.1358
FDR	0.012476	0.033942	0.033942	0.012476	0.012476	0.020761
P-value	2.58E-05	2.64E-04	2.64E-04	4.25E-05	4.32E-05	1.08E-04

**HeLa secretome**					
GO cellular component:	Lysosomal lumen	Vacuolar lumen	Primary lysosome	Azurophil granule	Vacuolar part
Enrichment ratio	65.506	43.983	31.213	31.213	17.529
FDR	1.24E-19	8.38E-21	8.75E-12	8.75E-12	5.62E-20
P-value	4.30E-22	7.25E-24	5.30E-14	5.30E-14	1.46E-22

**THP-1 secretome**					
GO cellular component:	Lysosomal lumen	Vacuolar lumen	Vesicle lumen	Secretory granule lumen	Lysosome
Enrichment ratio	40.106	22.176	13.673	13.090	9.3919
FDR	4.32E-09	2.03E-07	1.30E-06	1.30E-06	1.90E-07
P-value	3.74E-12	7.04E-10	1.01E-08	9.00E-09	4.93E-10

Supplementary Table 1. Gene Ontology (GO) enrichment analysis on proteomic data. GO was performed using WebGestalt overrepresentation analysis (ORA). P-values were calculated using a Fisher's exact test with a Benjamini-Hochberg (BH) correction for multiple comparisons. Input IDs were proteins identified as significantly enriched in proteomic data, as determined by a student's t-test ($FC > 1$, $p < 0.05$).
