# Supplementary material for: Coxiella burnetii manipulates the lysosomal protease cathepsin B to facilitate intracellular success
Source: Nat Commun. 2025 Apr 24;16:3844. doi: 10.1038/s41467-025-59283-3 (PMC12022341; doi:10.1038/s41467-025-59283-3)
Supplement: Supplementary file 2 — Description Of Additional Supplementary File [file 41467_2025_59283_MOESM2_ESM.pdf]

Description of Additional supplementary files

Supplementary Data 1. Mass spectrometry results. Raw files were searched in FragPipe (v.18.0) against the *H. sapiens* and *C. burnetii* reference proteomes. Resulting data files were further analysed using Perseus (v.1.6.0.7). Statistical comparison between groups was performed using a student's t-test with multiple hypothesis correction being applied based on FDR. Data are presented as Perseus output.

Supplementary Data 2. List of *C. burnetii* effector proteins screened for phenotypic effect on cathepsin B abundance. Cells were infected with *C. burnetii* mutant strains (transposon or deletion mutants) and subject to western blotting to determine if any strains led to cathepsin B retention. Alternatively, cells were transfected with plasmids encoding *C. burnetii* effectors and screened by immunofluorescence microscopy or western blotting to determine if expression of individual effectors led to cathepsin B removal.

Supplementary Data 3. List of strains, plasmids, and oligonucleotides used in this study.
