# Supplementary material for: Coxiella burnetii manipulates the lysosomal protease cathepsin B to facilitate intracellular success
Source: Nat Commun. 2025 Apr 24;16:3844. doi: 10.1038/s41467-025-59283-3 (PMC12022341; doi:10.1038/s41467-025-59283-3)
Supplement: Supplementary file 5 — Supplementary Data 3 [file 41467_2025_59283_MOESM5_ESM.docx]

**Table 1. List of strains, plasmids and oligonucleotides used in this study.**

| **Strain** | **Description** | **Source/reference** |
| --- | --- | --- |
| *C. burnetii* Nine Mile Phase II (NMII) | *C. burnetii* Nine Mile Phase II (NMII), RSA439, clone 4 (wild type) | Ted Hackstadt, Rocky Mountain Laboratories, USA |
| *C. burnetii* NMII *icmL*::Tn | WT *C. burnetii* with transposon inserted in *icmL* gene leading to non-functional type IV-B secretion system (Kan^R^) | ^1^ |
| *C. burnetii* NMII *icmS*::Tn | WT *C. burnetii* with transposon inserted in *icmS* gene (site 1582033) (Cm^R^) | ^1^ |
| *C. burnetii* NMII *0021*::Tn (*cvpB*::Tn) | WT *C. burnetii* with transposon inserted in *cbu_0021* gene (site 18798) (Cm^R^) | ^1^ |
| *C. burnetii* NMII *0021*::Tn + pJB-*CBU_0021-*3xFLAG | *C. burnetii CBU_0021*::Tn mutant (see above) complemented with expression plasmid containing 3xFLAG-*CBU_0021* | ^1^ |
| *C. burnetii* NMII *0021*::Tn + pJB-*CBU_0021*_306-809_*-*3xFLAG | *C. burnetii CBU_0021*::Tn mutant (see above) complemented with expression plasmid containing truncated *CBU_0021* (encoding amino acid 306-809) and C-terminal 3xFLAG tag | This study |
| *C. burnetii* NMII *0021*::Tn + pJB-*CBU_0021*_416-809_*-*3xFLAG | *C. burnetii CBU_0021*::Tn mutant (see above) complemented with expression plasmid containing truncated *CBU_0021* (encoding amino acid 416-809) and C-terminal 3xFLAG tag | This study |
| *C. burnetii* NMII *0021*::Tn + pJB-*CBU_0021*_603-809_*-*3xFLAG | *C. burnetii CBU_0021*::Tn mutant (see above) complemented with expression plasmid containing truncated *CBU_0021* (encoding amino acid 603-809) and C-terminal 3xFLAG tag | This study |
|  |  |  |
| **Plasmid** | **Description** | **Reference** |
| pLJM1-eGFP | Lentiviral expression vector for generation of stable cell lines. Used to generate HeLa-GFP only cells | Addgene #19319, ^2^ |
| pLJM1-CTSB-GFP | Lentiviral expression vector for generation of stable cell lines. Used to generate CTSB-GFP HeLa cells | This study |
| pLJM1-CTSB^C108A^-GFP | Lentiviral expression vector for generation of stable cell lines. Used to generate CTSB^C108A^-GFP HeLa cells | This study |
| pcDNA3.1-hCTSB | Mammalian expression of human cathepsin B | Addgene #11249, ^3^ |
| pVSV-g | Lentiviral packaging plasmid | Villadangos Lab,  Doherty Institute |
| pRSV-REV | Lentiviral packaging plasmid | Villadangos Lab,  Doherty Institute |
| pMDLg | Lentiviral packaging plasmid | Villadangos Lab,  Doherty Institute |
| pJB-Kan-3xFLAG-*CBU_0021*_306-809_ | *C. burnetii* expression plasmid for complementation of *CBU_0021* mutant with truncated *CBU_0021* (encoding amino acids 306-809) | This study |
| pJB-Kan-3xFLAG-*CBU_0021*_416-809_ | *C. burnetii* expression plasmid for complementation of *CBU_0021* mutant with truncated *CBU_0021* (encoding amino acids 416-809) | This study |
| pJB-Kan-3xFLAG-*CBU_0021*_603-809_ | *C. burnetii* expression plasmid for complementation of *CBU_0021* mutant with truncated *CBU_0021* (encoding amino acids 603-809) | This study |
|  |  |  |
| **Oligonucleotide** | **Sequence** | **Use/reference** |
| 18S rRNA qRT-PCR forward | CGGCTACCACATCCAAGGAA | qRT-PCR |
| 18S rRNA qRT-PCR reverse | GCTGGAATTACCGCGGCT | qRT-PCR |
| CTSB qRT-PCR forward | AGTGGAGAATGGCACACCCTA | qRT-PCR, targeted to CTSB heavy chain, ^4^ |
| CTSB qRT-PCR reverse | AAGAAGCCATTGTCACCCCA | qRT-PCR, targeted to CTSB heavy chain, ^4^ |
| CTSC qRT-PCR forward | TTACTGCAACGAGACAATGACTG | qRT-PCR, ^5^ |
| CTSB qRT-PCR reverse | AGGTGTGCTGTGTTGACATAC | qRT-PCR, ^5^ |
| CTSD qRT-PCR forward | AACTGCTGGACATCGCTTGCT | qRT-PCR, ^4^ |
| CTSD qRT-PCR reverse | CATTCTTCACGTAGGTGCTGGA | qRT-PCR, ^4^ |
| Forward primer to amplify cathepsin B (NheI) | AAAGCTAGCAATGTGGCAGCTCTGGGC | Generate lentiviral expression vector |
| Reverse primer to amplify cathepsin B (AgeI) | AAACCGGTAAGATCTTTTCCCAGTACTGATCGGTGC | Generate lentiviral expression vector |
| Site directed mutagenesis of cathepsin B (mutate cysteine 108 to alanine) | CAGGGCTCCTGTGGCAGCGCATGGGCCTTCGGGGCTGTG | Generate lentiviral expression vector |
| Site directed mutagenesis of cathepsin B (mutate cysteine 108 to alanine) | CACAGCCCCGAAGGCCCATGCGCTGCCACAGGAGCCCTG | Generate lentiviral expression vector |
| Forward primer to amplify *CBU_0021* at amino acid 306 (BamHI) | AAGGATCCATCCTTTGGGACAACTTACAAA | *CBU_0021*::Tn complementation construct |
| Forward primer to amplify *CBU_0021* at amino acid 461 (BamHI) | AAGGATCCATGATTAACCGATTGAATTTCCC | *CBU_0021*::Tn complementation construct |
| Forward primer to amplify *CBU_0021* at amino acid 603 (BamHI) | AAGGATCCATGCAAAATCTACTTCGCAATG | *CBU_0021*::Tn complementation construct |
| Reverse primer to amplify *CBU_0021* truncations (SalI) | GCGTCGACTTACTTAGTGAAAGAAGCAATGG | *CBU_0021*::Tn complementation construct |

**References:**

1. Newton, H. J. *et al.* A screen of *Coxiella burnetii* mutants reveals important roles for Dot/Icm effectors and host autophagy in vacuole biogenesis. *PLoS Pathog* **10**, e1004286 (2014). <https://doi.org/10.1371/journal.ppat.1004286>
2. Sancak, Y. *et al.* The Rag GTPases bind raptor and mediate amino acid signaling to mTORC1. *Science* **320**, 1496-1501 (2008). <https://doi.org/10.1126/science.1157535>
3. Huang, I. C. *et al.* SARS coronavirus, but not human coronavirus NL63, utilizes cathepsin L to infect ACE2-expressing cells. *J Biol Chem* **281**, 3198-3203 (2006). <https://doi.org/10.1074/jbc.M508381200>
4. Sardiello, M. *et al.* A gene network regulating lysosomal biogenesis and function. *Science* **325**, 473-477 (2009). <https://doi.org/10.1126/science.1174447>
5. Dang, C. *et al.* Identification of dysregulated genes in cutaneous squamous cell carcinoma. *Oncology reports* **16**, 513-519 (2006).
