# Supplementary material for: Coxiella burnetii manipulates the lysosomal protease cathepsin B to facilitate intracellular success
Source: Nat Commun. 2025 Apr 24;16:3844. doi: 10.1038/s41467-025-59283-3 (PMC12022341; doi:10.1038/s41467-025-59283-3)
Supplement: Supplementary file 6 — Reporting Summary [file 41467_2025_59283_MOESM6_ESM.pdf]

nature portfolio

Corresponding author(s): Hayley J Newton, Laura E Edgington-Mitchell

Last updated by author(s): Apr 9, 2025

Reporting Summary

Nature Portfolio wishes to improve the reproducibility of the work that we publish. This form provides structure for consistency and transparency in reporting. For further information on Nature Portfolio policies, see our Editorial Policies and the Editorial Policy Checklist.

Statistics

For all statistical analyses, confirm that the following items are present in the figure legend, table legend, main text, or Methods section.

n/a	Confirmed	
☐	☒	The exact sample size (*n*) for each experimental group/condition, given as a discrete number and unit of measurement
☐	☒	A statement on whether measurements were taken from distinct samples or whether the same sample was measured repeatedly
☐	☒	The statistical test(s) used AND whether they are one- or two-sided<br>*Only common tests should be described solely by name; describe more complex techniques in the Methods section.*
☒	☐	A description of all covariates tested
☐	☒	A description of any assumptions or corrections, such as tests of normality and adjustment for multiple comparisons
☐	☒	A full description of the statistical parameters including central tendency (e.g. means) or other basic estimates (e.g. regression coefficient) AND variation (e.g. standard deviation) or associated estimates of uncertainty (e.g. confidence intervals)
☐	☒	For null hypothesis testing, the test statistic (e.g. F, t, r) with confidence intervals, effect sizes, degrees of freedom and P value noted<br>*Give P values as exact values whenever suitable.*
☒	☐	For Bayesian analysis, information on the choice of priors and Markov chain Monte Carlo settings
☒	☐	For hierarchical and complex designs, identification of the appropriate level for tests and full reporting of outcomes
☒	☐	Estimates of effect sizes (e.g. Cohen's *d*, Pearson's *r*), indicating how they were calculated

Our web collection on statistics for biologists contains articles on many of the points above.

Software and code

Policy information about availability of computer code

Data collection	ImageLab 6.1 was used to acquire western blot images.
Data analysis	FIJI/ImageJ 2.1.0/1.53c was used for analysis of microscopy images. FragPipe v18.0 and Perseus v1.6.0.7 were used for analysis of proteomics data. RStudio (v4.2.1) was used for visualisation of proteomics data. GraphPad Prism 9 and Prism 10 were used for statistical analysis and data visualisation.

For manuscripts utilizing custom algorithms or software that are central to the research but not yet described in published literature, software must be made available to editors and reviewers. We strongly encourage code deposition in a community repository (e.g. GitHub). See the Nature Portfolio guidelines for submitting code & software for further information.

Data

Policy information about availability of data

All manuscripts must include a data availability statement. This statement should provide the following information, where applicable:

- Accession codes, unique identifiers, or web links for publicly available datasets
- A description of any restrictions on data availability
- For clinical datasets or third party data, please ensure that the statement adheres to our policy

The mass spectrometry proteomics data have been deposited to the ProteomeXchange Consortium via the PRIDE 72 partner repository with the dataset identifiers PXD052888, PXD052890, PXD052954 and PXD052955.

Research involving human participants, their data, or biological material

Policy information about studies with human participants or human data. See also policy information about sex, gender (identity/presentation), and sexual orientation and race, ethnicity and racism.

Reporting on sex and gender	*Use the terms sex (biological attribute) and gender (shaped by social and cultural circumstances) carefully in order to avoid confusing both terms. Indicate if findings apply to only one sex or gender; describe whether sex and gender were considered in study design; whether sex and/or gender was determined based on self-reporting or assigned and methods used. Provide in the source data disaggregated sex and gender data, where this information has been collected, and if consent has been obtained for sharing of individual-level data; provide overall numbers in this Reporting Summary. Please state if this information has not been collected. Report sex- and gender-based analyses where performed, justify reasons for lack of sex- and gender-based analysis.*
Reporting on race, ethnicity, or other socially relevant groupings	*Please specify the socially constructed or socially relevant categorization variable(s) used in your manuscript and explain why they were used. Please note that such variables should not be used as proxies for other socially constructed/relevant variables (for example, race or ethnicity should not be used as a proxy for socioeconomic status). Provide clear definitions of the relevant terms used, how they were provided (by the participants/respondents, the researchers, or third parties), and the method(s) used to classify people into the different categories (e.g. self-report, census or administrative data, social media data, etc.) Please provide details about how you controlled for confounding variables in your analyses.*
Population characteristics	*Describe the covariate-relevant population characteristics of the human research participants (e.g. age, genotypic information, past and current diagnosis and treatment categories). If you filled out the behavioural & social sciences study design questions and have nothing to add here, write "See above."*
Recruitment	*Describe how participants were recruited. Outline any potential self-selection bias or other biases that may be present and how these are likely to impact results.*
Ethics oversight	*Identify the organization(s) that approved the study protocol.*

Note that full information on the approval of the study protocol must also be provided in the manuscript.

Field-specific reporting

Please select the one below that is the best fit for your research. If you are not sure, read the appropriate sections before making your selection.

☒ Life sciences ☐ Behavioural & social sciences ☐ Ecological, evolutionary & environmental sciences

For a reference copy of the document with all sections, see nature.com/documents/nr-reporting-summary-flat.pdf

Life sciences study design

All studies must disclose on these points even when the disclosure is negative.

Sample size	No statistical methods were used to predetermine sample size. Sample sizes were chosen based on how many samples could feasibly be processed in the given time frame or were previously determined in previous studies (see Newton et al., PNAS 2020 (DOI: 10.1073/pnas.1921344117; Lau et al., Mol Micro 2022 (DOI: 10.1111/mmi.14858)).
Data exclusions	For all proteomics analysis, data were filtered to require a protein to be identified (ie give a non-zero value) in 3/4 replicates from at least one condition tested.
Replication	Western blots were performed at least 3 times to ensure reproducibility of results, noted in figure legends and individual blots available in source data file.
Randomization	Samples were randomly allocated into respective conditions (ie infected/uninfected)
Blinding	For quantification of microscopy data on CCV size, other lab members numbered samples prior to quantification so that the imaging and quantification was done blinded.

Reporting for specific materials, systems and methods

We require information from authors about some types of materials, experimental systems and methods used in many studies. Here, indicate whether each material, system or method listed is relevant to your study. If you are not sure if a list item applies to your research, read the appropriate section before selecting a response.

Materials & experimental systems

n/a	Involved in the study	
☐	☒	Antibodies
☐	☒	Eukaryotic cell lines
☒	☐	Palaeontology and archaeology
☒	☐	Animals and other organisms
☒	☐	Clinical data
☒	☐	Dual use research of concern
☒	☐	Plants

Methods

n/a	Involved in the study	
☒	☐	ChIP-seq
☒	☐	Flow cytometry
☒	☐	MRI-based neuroimaging

Antibodies

Antibodies used	The following antibodies were used for western blotting (WB) or immunofluorescence microscopy (IF): anti-cathepsin B monoclonal (Cell Signalling Technology #31718, WB 1:2000, IF: 1:200), anti-cathepsin B polyclonal (R&D Systems, #AF965, WB 1:1000), anti-cathepsin C (Santa Cruz Biotechnology #sc-74590, WB 1:2000), anti-cathepsin D (Abcam #ab72915, WB 1:2000), anti-DotB (Edward Shaw Laboratory, WB 1:2000), anti- -actin (Sigma Aldrich #A1978, WB 1:8000), anti-3xFLAG (Sigma, #F3165, IF 1:250) anti-LAMP-1 (Developmental Studies Hybridoma Bank #H4A3, IF 1:250), anti-Coxiella burnetii (Craig Roy Laboratory, IF 1:10,000), anti-LC3B (Novus, #NB100-2220, WB 1:2000), anti-CI-M6PR (Novus #NB300-514, IF 1:100), anti-Ubiquitin (Cell Signalling Technology #3936S, WB 1:2000).
Validation	Antibodies were selected based on literature searches for previous publications demonstrating use of the antibody and/or manufacturer provided validation. Anti-cathepsin B monoclonal (Cell Signalling Technology #31718) has been cited extensively (see Yang et al., iScience 2024 (DOI: 10.1016/j.isci.2024.111024), Guiterrez-Ruiz et al., Cell Rep 2023 (DOI: 10.1016/j.celrep.2023.113042) amongst others and has been validated for use in immunoblotting and microscopy by the manufacturer. Anti-cathepsin B polyclonal (R&D Systems #AF965) was previously used in Xu et al., BiolChem 2024 (doi.org/10.1515/hsz-2023-0355), Edgington-Mitchell et al., Oncotarget 2015 (doi: 10.18632/oncotarget.4714). Anti-cathepsin C (Santa Cruz Biotechnology #sc-74590) has been used previously (Ze Wu et al., 2023 J Cell Bio (doi: 10.1083/jcb.202208155), Wang et al., Dev Cell 2023 (doi: 10.1016/j.devcel.2023.03.014) and is validated by the manufacturer for use in western blotting. Anti-cathepsin D (abcam #ab72915) was used previously (Xu et al., Autophagy 2019, (doi: 10.1080/15548627.2019.1569928) and is validated for use in microscopy and western blot by the manufacturer on their website. Anti-DotB was synthesised in the laboratory of Prof Edward Shaw and has been used previously (Kuba et al., 2020 Infect Immun (doi: 10.1128/IAI.00913-19). Anti-B-actin (Sigma #A1978) has been used extensively (>4000 citations on Sigma website). Anti-3xFLAG (Sigma #F3165) has also been used extensively (>8000 citations on Sigma website). Anti-LAMP1 (DSHB #H4A3) has been used previously (Lau et al., 2019 Mol Microbiol (doi: 10.1111/mmi.14858), Newton et al., 2020 PNAS (doi: 10.1073/pnas.1921344117). Anti-Coxiella burnetii was synthesised in the lab of Professor Craig Roy (Yale University) and has been used previously (Crabill et al., 2019 Infect Immun (doi: 10.1128/IAI.00758-17), Newton et al., 2014 PloS Path (doi: 10.1371/journal.ppat.1004286). anti-LC3B (Novus #NB-100-2220) has been used previously (Lau et al., 2019 Mol Microbiol (doi: 10.1111/mmi.14858), Tran et al., Commun Biol 2024 (DOI: 10.1038/s42003-024-05890-7) amongst many others (>1000 citations). Anti-CI-M6PR (Novus #NB300-514) has been cited previously (Calcagni et al., 2023 Nat Commun (DOI: 10.1038/s41467-023-39643-7) and is validated on their website for both IF and WB. Anti-Ubiquitin (Cell Signalling Technology #3936S) has been used extensively (see Luo et al., 2025 Nat Commun (doi: 10.1038/s41467-025-56570-x) amongst others (>1000 citations).

Eukaryotic cell lines

Policy information about cell lines and Sex and Gender in Research

Cell line source(s)	HeLa CCL2 (cervical epithelial), HEK293T (embryonic kidney) and THP-1 cells (monocyte) were sourced from ATCC.
Authentication	ATCC comprehensively performs authentication and quality-control tests on all distribution lots of all cell lines.
Mycoplasma contamination	All parental cell lines were tested for mycoplasma contamination. Stable cell lines generated in this study were made from mycoplasma-negative parental cells.
Commonly misidentified lines (See ICLAC register)	No misidentified lines were used.

Plants

Seed stocks	*Report on the source of all seed stocks or other plant material used. If applicable, state the seed stock centre and catalogue number. If plant specimens were collected from the field, describe the collection location, date and sampling procedures.*
Novel plant genotypes	*Describe the methods by which all novel plant genotypes were produced. This includes those generated by transgenic approaches, gene editing, chemical/radiation-based mutagenesis and hybridization. For transgenic lines, describe the transformation method, the number of independent lines analyzed and the generation upon which experiments were performed. For gene-edited lines, describe the editor used, the endogenous sequence targeted for editing, the targeting guide RNA sequence (if applicable) and how the editor was applied.*
Authentication	*Describe any authentication procedures for each seed stock used or novel genotype generated. Describe any experiments used to assess the effect of a mutation and, where applicable, how potential secondary effects (e.g. second site T-DNA insertions, mosiacism, off-target gene editing) were examined.*
